# Observation of laser pulse propagation in optical fibers with a SPAD camera

**DOI:** 10.1038/srep43302

**Published:** 2017-03-07

**Authors:** Ryan Warburton, Constantin Aniculaesei, Matteo Clerici, Yoann Altmann, Genevieve Gariepy, Richard McCracken, Derryck Reid, Steve McLaughlin, Marco Petrovich, John Hayes, Robert Henderson, Daniele Faccio, Jonathan Leach

**Affiliations:** 1 Institute of Photonics and Quantum Sciences, Heriot-Watt University, Edinburgh, EH14 4AS, UK; 2 Center for Relativistic Laser Science, Institute for Basic Science (IBS), Gwangju 61005, Republic of Korea; 3 School of Engineering, University of Glasgow, Glasgow, G12 8LT, UK; 4 Institute of Sensors, Signals and Systems, Heriot-Watt University, Edinburgh, EH14 4AS, UK; 5 Optoelectronics Research Centre, University of Southampton, Highfield, Southampton, SO17 1BJ Hampshire; 6 Institute for Micro and Nano Systems, University of Edinburgh, Alexander Crum Brown Road, Edinburgh EH9 3FF, UK

## Abstract

Recording processes and events that occur on sub-nanosecond timescales poses a difficult challenge. Conventional ultrafast imaging techniques often rely on long data collection times, which can be due to limited device sensitivity and/or the requirement of scanning the detection system to form an image. In this work, we use a single-photon avalanche detector array camera with pico-second timing accuracy to detect photons scattered by the cladding in optical fibers. We use this method to film supercontinuum generation and track a GHz pulse train in optical fibers. We also show how the limited spatial resolution of the array can be improved with computational imaging. The single-photon sensitivity of the camera and the absence of scanning the detection system results in short total acquisition times, as low as a few seconds depending on light levels. Our results allow us to calculate the group index of different wavelength bands within the supercontinuum generation process. This technology can be applied to a range of applications, e.g., the characterization of ultrafast processes, time-resolved fluorescence imaging, three-dimensional depth imaging, and tracking hidden objects around a corner.

One of the first slow motion events captured on film was a galloping horse. “Sallie Gardner at a Gallop” is a series of 24 photographs taken in rapid succession to analyse the gait of a horse^1^. It demonstrated that all four feet were simultaneously off the ground during the gallop. Ever since, there has been a fascination with slow-motion video, and by the 1930s, speeds of 1000 frames per second (fps) were achievable on 16 mm film with a built-in shutter/correction plate^2^. By the early 1960s, a 10,000 fps rotating prism camera was demonstrated^3^. Technological developments have led to cameras capable of realtime monochromatic filming at several million fps^4^ and the observation of femtosecond pulses of light using a streak camera combined with a stroboscopic approach^5^. Most recently, compressed single-shot photography at 100 billion frames per second was reported using a streak camera^6^ without relying on stroboscopic illumination. The main limitations of this work were the limit of 350 frames per acquisition (3.5 ns) and the need for computational reconstruction techniques to realise a final video.

In photon-starved applications, single-photon detection and, more specifically, time-correlated single-photon counting (TCSPC), offers extreme sensitivity and picosecond timing resolution^7^. For many applications, single-photon avalanche diodes (SPADs) are the favoured choice of detector due to their relatively compact nature, ease of integration, high efficiency and low noise characteristics, combining to give ultra-high sensitivity devices. It is common to use silicon SPADs for visible wavelengths^8^ and InGaAs/InP SPADs for NIR and telecommunications wavelengths^9^. When coupled to an optical system, usually with optical fiber, SPADs offer single-point detection. While useful in many applications, such as time-resolved photoluminesence^10^, single-point detection imposes limitations on measurements where photons need to be collected from a number of discrete spatial positions such as single-photon LIDAR^11^. To alleviate this, the outgoing beam can be scanned to form an image^12^ through the use of galvonometer mirrors to raster-scan both the outgoing beam and the returned photons to the collection fiber. This increases acquisition times since all spatial positions cannot be measured simultaneously. However, it has been shown that per-pixel dwell times of just a few milliseconds can yield sufficient signal to recreate a depth image of the scene at 325 m standoff distance^13^.

Recent advances in CMOS processing have enabled the integration of control and counting electronics on the same chip as the SPAD itself^14^. Here, the SPAD and associated electronics form a “smart pixel”. These smart pixels can be processed into an array to form a 2D sensor of SPADs, each with its own timing electronics capable of picosecond timing resolution^15^. This technology has been used previously for fluorescence lifetime imaging^16^, sub-Rayleigh imaging^17^, and for ranging and 3D reconstruction with an external time to digital converter (TDC)^18^. These techniques have been extended to applications such as detecting objects hidden from view^19^,^20^ and tracking moving objects around a corner^21^. Recently, light-in-flight imaging was implemented to visualize a laser pulse propagating in air^22^.

In this work, we use a SPAD camera with a temporal instrument response function (IRF) of ~150 ps in each pixel to record and examine ultrafast processes in optical fibers. We report two main results: first, the observation of supercontinuum generation in photon crystal fiber (PCF) at a repetition rate of 80 MHz to investigate the evolution of the spectrum generation; and second, the observation of a train of femtosecond pulses at 803 nm at a repetition rate of 1 GHz to test the limit of the frequency at which the camera can operate. We also develop a model for the observed data, which allows us to increase the spatial resolution for the reported results.

## Results

### Supercontinuum generation in PCF

Supercontinuum generation in the 400–700 nm wavelength range was first demonstrated in 1970 through four-wave mixing in borosilicate glass^23^. Since then, many new techniques have been devised to create supercontinuum sources, with the most efficient and useable techniques favouring the use of high energy ultrashort pulse lasers and PCF^24^. The processes involved in the generation of a broad spectrum from a monochromatic pump are well known and well documented in the literature. There are also videos and photographs of increasing spectral broadening with increasing pump intensity. Here, we record the generation of a supercontinuum by collecting photons scattered from the fiber cladding with a SPAD camera, enabling the visualization of discrete wavelength ranges through the use of bandpass interference filters.

**Figure 1:**
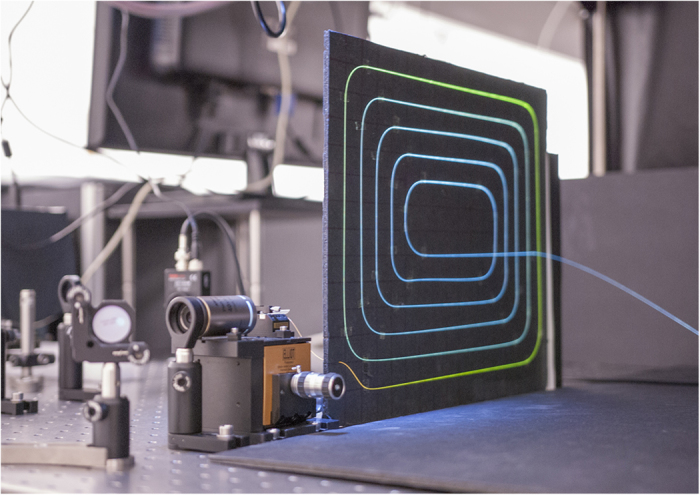
Photograph of the PCF and supercontinuum generation. A 40x microscope objective couples the 130 fs pulses at a wavelength of 780 nm pulses from the Coherent Chameleon laser into the PCF. The PCF is arranged in a spiral to allow the SPAD to view a long propagation length despite its relatively small field of view. Red, orange, and green wavelengths are generated after a short propagation distance, whereas higher energy wavelengths appear further along the fiber length.

A Coherent Chameleon laser, emitting 130 fs pulses at 780 nm at a frequency of 80 MHz was coupled into the PCF (3.4 *μ*m core, where the microstructured cladding had a 5.3 *μ*m pitch and relative hole size of 0.88 provided by the ORC, Southampton) using a x40 microscope objective as shown in Fig. 1. Since the SPAD camera has a relatively small field of view (~30 cm square at a standoff distance of 1.5 m), the fiber was arranged in a spiral pattern to maximise the propagation length visible to camera. Due to the length of the fibre within the field of view, the refractive index of the fiber, and the pulse separation of 12.5 ns, there are sometimes 2 pulses visible in the fiber. The power of the laser/coupling efficiency into the PCF was kept constant throughout the measurements since we expect the nonlinearity induced change in dispersion to vary with input power.

To observe the generation of certain wavelength ranges separately, narrow bandpass interference filters were placed in front of the SPAD array. The central wavelengths of each filter were: 450, 500, 550, 600 and 650 nm, each with a FWHM bandwidth of 40 nm. Data was acquired for 300s (1 million frames, 100 us per exposure) for each filter.

**Figure 2:**
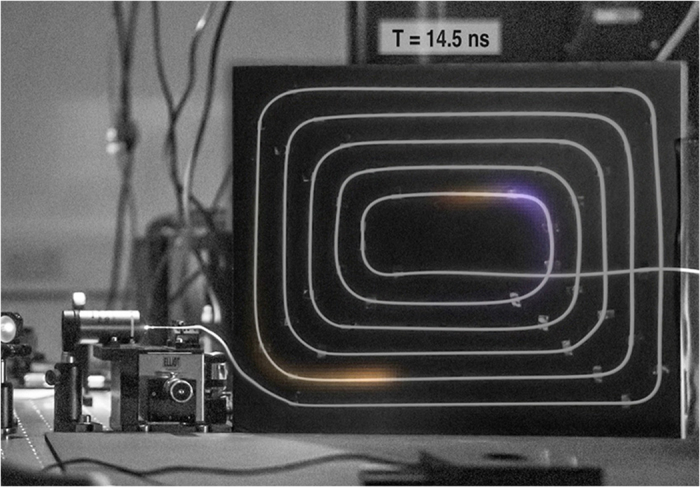
A single frame from video 6 in the supplementary information showing supercontinuum generation in PCF. All signals from the 6 separate wavelength bands were combined to show the supercontinuum generation in the visible. At t = 14.5 ns, the outermost pulse contains no blue light, whereas the innermost pulse that has propagated through the entire length of the fiber contains a strong blue component. This blue component also appears to be slightly delayed with respect to the red/orange components.

Supplementary Videos 1,2,3,4,5,6 show results for supercontinuum generation in PCF. It is interesting to note that not all wavelengths are generated at the same point along the fiber as can be observed in Fig. 1; green (500/550 nm), orange (600 nm) and red (650 nm) are generated first, whilst blue (450 nm) starts to appear after a propagation distance of approximately 1 m which is an effect particular to this specific fiber. Supplementary Videos 1,2,3,4,5, relating to 450, 500, 550, 600 and 650 nm, respectively, show this effect clearly. All the videos (and the frames extracted from these) were created by overlaying colorized data fitted from the model on top of an image of the fibers used *in*-*situ* with a regular digital camera. Video 6 shows the supercontinuum generation evolution by overlaying the data from each wavelength range to show the full spectrum measured using the SPAD camera. A single frame from video 6 was extracted and is shown in Fig. 2.

From Fig. 2, it can be seen that the blue component of the generated supercontinuum is delayed with respect to the red, orange and green components. This introduces other aspects of the supercontinuum generation that can be qualitatively investigated using the timing information from the SPAD camera. By analysing each wavelength range independently, the blue (450 nm) pulse was delayed by approximately 360 ps with respect to the red (600 nm) pulse after propagation through the full length of the PCF (410 cm). This demonstration opens up applications where, for example, fibers or components that are installed in systems need to be analysed non-invasively.

**Figure 3:**
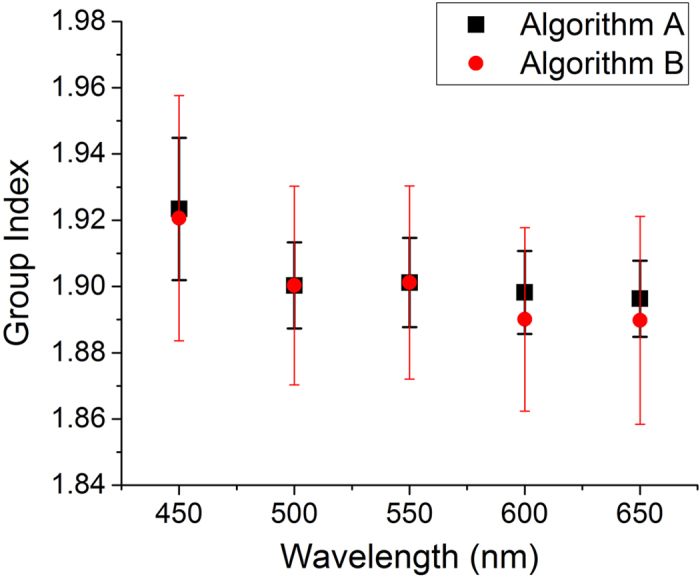
Estimated group index vs. wavelength of five separate wavelength bands of the supercontinuum generated in the PCF, measured using the SPAD camera. The data was analysed with two separate algorithms; Algorithm A is described in the text below and Algorithm B can be found in the supplementary information.

Using two different algorithms for data analysis (Algorithm A is described in the body of the paper and Algorithm B is explained in the supplementary information), a qualitative estimate of the group index for each wavelength range was extracted; these data are plotted in Fig. 3. Both algorithms show the same trend: a higher group index is measured for the blue component in the PCF, whereas longer wavelengths have a lower group index. The main source of uncertainty with regards to the group velocity is the slight variation in IRF for each of the SPAD array pixels, i.e. the peak photon return time of the IRF and the width of the IRF are not constant across the whole array when illuminated with a plane wave. We note that direct measurements of the group index are important for multimode fibres, such as that characterized here, since models of idealized structures may not be able to capture the full details of the intra-mode coupling (due, for example, to input coupling conditions and bending of the fiber). The intra-mode coupling or mode distribution will modify the group index and overall propagation dynamics of ultrashort laser pulses. Our method, although only qualitative due to the relatively large error bars, measures the average group index regardless of modal distribution in the fiber and also captures the local (i.e. at a fixed position along the fiber) distribution of the light pulse.

### 1 GHz pulse train

The maximum sychronization rate accepted by the internal clock of the SPAD camera is 100 MHz. For frequencies greater than this it is still possible to perform measurements, however within the timing histogram there will be multiple peaks corresponding to pulses for which the camera was not able to accept a trigger signal. If required, this can be addressed with post-processing of the data so that the timing data is divided into sections that correspond to the clock period and summed^25^. In this section, we record a number of individual pulses propagating through an optical fiber at a frequency of 1 GHz, meaning the pulses are separated by only 1 ns yet still fully resolvable by the SPAD camera.

The laser (Gigajet by Laser Quantum) emits 35 fs pulses at 803 nm at a frequency of 1 GHz. As with the previous setup, the beam is sampled and sent to a fast APD (Thorlabs APD210). The signal from the APD is then divided down 32 times and sent through a comparator to achieve a TTL pulse at a repetition rate suitable for the SPAD camera. The laser was coupled into a Corning Fibrance optical fiber. These fibers are designed to scatter light from the core and are intentionally lossy. Whilst this is not ideal for propagation distance, it reduces acquisition times for filming the pulse train since more photons are scattered towards the camera.

**Figure 4:**
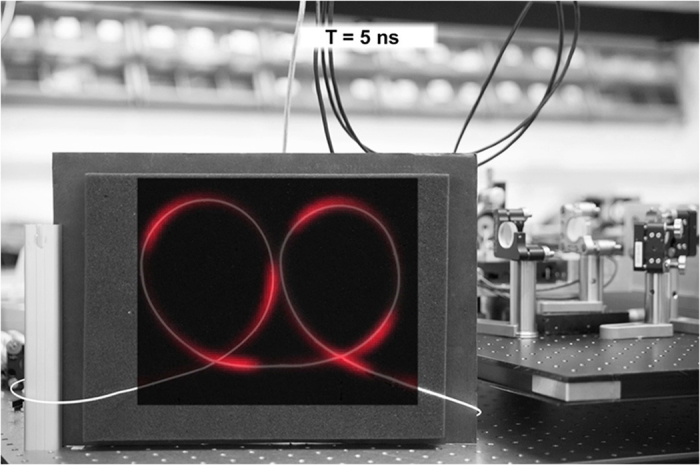
A single frame from the supplementary material video 7, showing multiple individual pulses at 780 nm propagating through the optical fiber with a repetition frequency of 1 GHz corresponding to a pulse separation of 1 ns. The processed data from the the camera is overlaid on top of a digital camera still image of the fiber *in*-*situ*.

In Fig. 4, in a single video frame, there are multiple laser pulses present due to the down-division of the 1 GHz trigger signal to satisfy the camera triggering limitation. Supplementary Video 7 shows the propagation of multiple pulses through the fiber overlaid on top of a digital camera photography of the fiber *in*-*situ*.

### Data Processing

We use a data processing algorithm that allows us to both denoise the data and generate high resolution images of the pulse propagation. The SPAD camera records three dimensional data (i.e., two spatial and one temporal dimensions) associated with the observed phenomena. Each SPAD element of the array operates in time-correlated single-photon counting (TCSPC) mode and can produce a histogram of the photon arrival times. In the first instance, it is possible to visualize the 3D data as a movie/sequence of images, where each frame shows the spatially resolved photon flux density associated with a given timing bin, defined as the temporal resolution of the TCSPC electronics. However, the quality of the recorded data is intrinsically limited by the low spatial resolution of the available arrays (e.g., 32 × 32 pixels in the reported experiments), by the low signal to noise ratio resulting from short acquisitions (the signal to noise ratio increases with the acquisition time) and additional sources of noise (e.g., ambient light and dark-count rates). The quality of the data can also be affected by the heterogeneity of the SPAD elements (heterogeneous sensitivity, presence of “hot-pixels”). Depending on the underlying observed phenomena, it is however possible to derive signal processing strategies to enhance the data quality and extract information of interest. In this section, we report the statistical model and associated signal processing method we developed for applications where the spatially resolved dynamics can be translated into an object detection/tracking problem. Here we consider tracking laser pulses propagating through a fibre. Assuming the fibre is included in a 2D plane (i.e., by neglecting the spatial dimension orthogonal to the pulse propagation), the tracking problem reduces to identifying the trajectory of each pulse in a two dimensional space.

#### Observation model

After collecting sufficient data, the camera outputs a matrix **D** with dimensions 1024 × *T*, where the columns correspond to the spatial intensity as a function of *x* and *y* at a given time *t*, and *T* is the total number of frames. When playing back the data in video form, the matrix **D** is reformatted into a data cube of dimensions 32 × 32 × *T*, where the first two dimensions correspond to a frame of a movie at a given time. To increase the low spatial resolution of these data, we construct a model of the light propagation in the fibers and find a best fit to the measured data. The model provides a matrix **I**, with the same dimensions as the data collected from the camera **D**, that can be reformatted in the same manner. The best fit parameters include the trajectory of the pulses as a function of time, and using these parameters, we can generate images with high spatial and temporal resolution of the pulse propagation.

First, we consider the intensity *I*(*x, y, t*) of one frame of the final movie. Assuming that a single peak is observed, this can be modelled as the convolution of a pulse *p*(*x, y, t*) with the impulse response function (IRF) of the camera *g*(*t*)


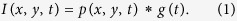


The function *p*(*x, y, t*) is the intensity distribution of a pulse with a 2D Gaussian profile at spatial position *x, y* at time *t*


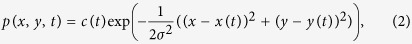


where *σ*^2^ controls the spatial dispersion, and *c*(*t*) is the amplitude of the pulse, which can vary with time, and the trajectory of a laser pulse is expressed as the vector **x**(*t*) = [*x*(*t*), *y*(*t*)]^*T*^. We find that an inverse-gamma probability density function provides a good approximation of the IRF and models the “blurring” introduced to the pulses’ paths well. Here, the parameters that define the inverse-gamma probability function are fixed, such that the amount of blurring at the start of the fiber is equal to that at the end. As mentioned previously, the IRF of the camera is ~150 ps. Comparing this to input laser temporal duration of 130 fs, we assume that the biggest contribution to the system IRF is from the SPAD detectors themselves. Hence, the system IRF is set to be constant throughout the fiber length. When we fit the model to the data, we need to match the dimensions of *I*(*x, y, t*) to the spatial resolution of the camera. We therefore evaluate the function *I*(*x, y, t*) on a 32 × 32 grid for the purposes of fitting. However, after the fitting and once the parameters of the trajectory are known, we are able to evaluate *I*(*x, y, t*) at much higher resolutions.

Second, we consider the intensity *I*(*x, y, t*) at all of the recorded times; this is the matrix **I**, which has dimensions 1024 × *T*. Following from equation 1, this is equal to


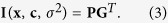


Here, **P** is a 1024 × *T* matrix corresponding to the pulse location and intensity for all times, and **G**^*T*^ is a *T* × *T* matrix that models the temporal dispersion of the pulse. The columns of **P** correspond to the re-shaped matrices *p*(*x, y, t*); the first dimension of **P** represents the spatial dimensions (*x* and *y*) and second dimension represents the temporal one (*t*). The matrix **I** corresponds to the data that the camera generates by recording the path of one pulse of light.

Finally, to include multiple pulses in the model, we assume that the laser source produces identical pulses and that all pulses follow the same trajectory and have the same velocity. Therefore the positions/trajectory and amplitude of the *k*^*th*^ pulse **x**_*k*_ can be expressed as a function of the zeroth pulse **x**_0_ as 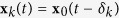 and 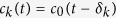, where *δ*_*k*_ is a temporal delay between the *k*^*th*^ and reference pulses. Thus, tracking the *K* observed pulses reduces to estimating the amplitudes and trajectory of a single pulse and the delay between that pulse and the (*K* − 1) other pulses. Assuming that the global intensity field produced by the *K* pulses can be explained by the sum of their individual contributions, the intensity profile can be expressed as


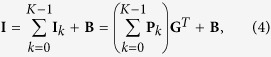


where 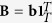 represents the background. The vector **b** has length 1024 and models the background intensities of the pixels (e.g., ambient light and detector noise) and is assumed to be constant over the acquisition time (**1**_*T*_ denotes a vector of ones of length *T*).

The noise corrupting the data is modelled by Poisson noise whose signal-dependent mean is given by (4). This is due to the nature of the recorded image sequences (histograms of detected photons) and the short acquisition time considered. This Poisson noise assumption has been assessed and validated from preliminary recordings by turning off the laser source (and detecting only background and dark-count photons). The next section details the proposed method to estimate the unknown model parameters. It is interesting that this estimation problem can be viewed as a multiple object tracking problem embedded within a denoising (due to the presence of Poisson noise) and deconvolution problem (due to *g*(·)).

#### Estimation strategy

Due to relative low spatial resolution of the recorded images and the limited acquisition time, we use a Bayesian approach to solve the tracking problem. The proposed Bayesian framework provides an elegant and powerful set of tools to combine the observed data with additional information available about the problem (e.g., background levels, laser source freqency, …) through so-called prior distributions. In order to obtain tractable analytical expressions for the pulses trajectory and amplitude, *x*_0_(*t*), *y*_0_(*t*) and *c*_0_(*t*) are approximated by finite series of Gaussian peaks whose weights control the temporal variations of the pulse trajectory and amplitude. Finally, we develop a Markov chain Monte Carlo (MCMC) inference method to estimate the unknown model parameters, namely, the weights associated with the reference pulse trajectory and amplitude, the delays {*δ*_*k*_}_*k*_ between the reference and other laser pulses and the spatial dispersion of the pulses *σ*^2^ in (2). More precisely, the prior distributions assigned to the unknown model parameters are first combined with the observation model (likelihood) to derive the joint posterior distribution of these parameters. Unfortunately, the complexity of the resulting posterior distribution (mainly due to the observation model complexity) does not allow the use of classical optimization techniques to estimate the parameters of interest (convergence issues). To tackle this problem we use an MCMC method to generate random variables according to that posterior distribution. The generated samples are finally used to approximate classical *minimum mean square error* (MMSE) estimator^26^. In addition to better convergence properties compared to classical optimization methods, the proposed strategy also allows the derivation of measures of uncertainty about the estimated parameters which can be used to assess the relevance of the model and the quality of the data.

Once the trajectory (i.e., the weights involved in the decomposition of the trajectory) has been estimated, we can analytically derive **x**_0_(*t*) to estimate the instantaneous pulse group velocity for each frame via 
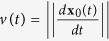
. Assuming that the group velocity is constant upon the propagation, it can finally be estimated by averaging over the *T* frames.

Thanks to the analytical expression of the observation model, it is straightforward to produce an enhanced image sequence, (without Poisson noise and background counts) using (4) and the estimated parameter values. More interestingly, it is also possible to produce higher spatial resolution images by considering finer sampling grids in (2). Videos 1,2,3,4,5,6,7 in the supplementary information show examples of enhanced 128 × 128 pixels images. It should also be noted that we could also improve the temporal resolution if needed.

## Discussion

Cameras built upon SPAD arrays are rapidly changing ultra-fast imaging methods. Their picosecond temporal resolution enables such cameras to record ultrafast processes, and due to their single-photon sensitivity, they are well suited to photon-starved applications.

In this work, we have used a SPAD camera operating in TCSPC mode to observe the propagation of laser pulses in optical fibers by capturing photons scattered from the fiber cladding. We observe supercontinuum generation in PCF and estimate the group indices for different wavelengths without direct access to the fiber. One possible application of this technology is the identification of imperfections or damage within optical fibers at a standoff distance. The ease of integrating optical bandpass filters in the camera and the sensitivity to ultra-low intensities also opens up the ability to record fluorescence spectra over a large range of wavelengths. The tracking of GHz pulses could be useful for measuring inter-pulse jitter from high frequency sources. In this scenario, the camera is triggered by one laser pulse but observes multiple pulses with the timing window. A laser with a high inter-pulse jitter will show a broadening of the temporal response as measured by the SPAD camera.

Many factors contribute to the minimum input laser power required to achieve pulse visualization in optical fibers, and the methods herein will not always be suitable for every application. These factors include: number of scattering sites within the optical fiber under test; standoff distance to fiber (since light is scattered in all directions from the fiber, the greater the standoff distance, the lower the signal according to the inverse square law); collection optics (a low f-number system will help collect photons but may increase jitter due to the high NA); and the fill factor of the SPAD array which will increase in newer iterations of the camera. However, as the SPAD camera is one of the most sensitive detectors, we anticipate that very low scattering events can be recorded^22^.

One limitation of the SPAD camera is that it relies upon recording a process many thousands of times to build up accurate statistics of photon arrival times, as with all TCSPC techniques. However, single-shot imaging techniques have been developed^5^, and the implementation of such a method with a SPAD camera is of interest for future work.

## Methods

**Figure 5:**
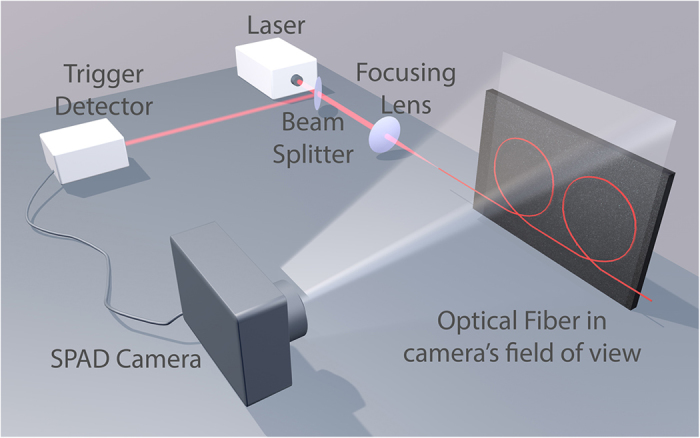
Schematic diagram of the setup. The laser emits pulses that are coupled into a an optical fiber that is in the field of view of the SPAD camera. Photons that are scattered from the optical fibre are then detected by the SPAD camera. A small fraction of the beam power is sampled with the beam splitter for the optical trigger. The optical trigger provides a periodic synchronization pulse for the SPAD camera in order to record time-correlated arrival times of the photons scattered from the optical fiber.

A schematic of the experimental setup is depicted in Fig. 5. The field of view of the camera is defined by the imaging lens (Samyang 8 mm f/3.5 fisheye lens), and the size of the SPAD array (1.6 × 1.6 mm) in the focal plane of the lens. The camera was positioned such that the field of view of the camera encompasses the optical fiber through which the pulses were propagating to maximise the spatial resolution of the camera. Before the laser is coupled into the optical fibre, a beamsplitter is used to sample the beam. For the supercontinuum measurements, this portion of the beam is incident on an optical constant fraction discriminator (OCF-401 from B&H), the output pulse of which acts as the periodic synchonization pulse for the timing electronics of the SPAD camera. In the case of the GHz pulse train measurements, a Thorlabs fast APD (APD210) was used to provide the synchronization. Photons scattered from the fiber (Corning Fibrance for the 1 GHz pulse train measurements and a 3.4 *μ*m core PCF for the supercontinuum) are detected by the SPAD camera, which is operated in time correlated single-photon counting (TCSPC) mode.

In order to calculate and calibrate our measurements of group index, we measured the group index at 532 nm using photodiodes to record the transit time of a pulse through a predefined length (10 m) of identical PCF fiber. The result of this measurement gave a group index of 1.9 ± 0.03. From this, the length of the fiber used in the supercontinuum generation measurements was ascertained, and subsequently the group indices at the wavelengths shown in Fig. 3 were calculated.

The PCF was fabricated using a standard two-stage stack and draw technique using commercial Suprasil F300 from Heraeus. The solid core (3.4 *μ*m) is surrounded by 5 rings of uniform holes with a spacing of 5.3 *μ*m and a relative hole size of 0.88.

### Time correlated single-photon counting

The TCSPC electronics of each pixel of the array is operated in reverse start-stop mode- the timer is started by the detection of a single photon, and stopped by the arrival of a periodic trigger from the OCF-401 for the supercontinuum measurements, and a Thorlabs fast APD (APD210) in the GHz pulse train measurements. The time difference between these start-stop events is recorded and read out through an FPGA to a computer via USB where they are stored in histogram form to build up accurate statistics of the arrival time of the photons for each pixel.The histogram timing window in TCSCP mode is ~55 ns with a bin-width of 54 ps, and the median dark count rate of the array is 50 counts per second per pixel.

### Data Availability

http://dx.doi.org/10.17861/da2e7605-4c31-4f04-9bba-eaac907add96.

## Additional Information

**How to cite this article**: Warburton, R. *et al*. Observation of laser pulse propagation in optical fibers with a SPAD camera. *Sci. Rep.* **7**, 43302; doi: 10.1038/srep43302 (2017).

**Publisher's note:** Springer Nature remains neutral with regard to jurisdictional claims in published maps and institutional affiliations.

## Supplementary Material

Supplementary video 1

Supplementary video 2

Supplementary video 3

Supplementary video 4

Supplementary video 5

Supplementary video 6

Supplementary video 7

Supplementary Information
