# Supplementary material for: Observation of laser pulse propagation in optical fibers with a SPAD camera
Source: Sci Rep. 2017 Mar 7;7:43302. doi: 10.1038/srep43302 (PMC5339868; doi:10.1038/srep43302)
Supplement: Supplementary Information [file srep43302-s8.pdf]

Observation of laser pulse propagation in optical fibers with a SPAD camera: SUPPLEMENTARY INFORMATION

Ryan Warburton[1,*], Constantin Aniculaesei[1,2], Matteo Clerici[1,3], Yoann Altmann[4], Genevieve Gariepy[1], Richard McCracken[1], Derryck Reid[1], Steve McLaughlin[4], Marco Petrovich[5], John Hayes[5], Robert Henderson[6], Daniele Faccio[1], and Jonathan Leach[1]

[1]Institute of Photonics and Quantum Sciences, Heriot-Watt University, Edinburgh, EH14 4AS, UK
[2]Center for Relativistic Laser Science, Institute for Basic Science (IBS), Gwangju 61005, Republic of Korea
[3]School of Engineering, University of Glasgow, Glasgow, G12 8LT, UK
[4]Institute of Sensors, Signals and Systems, Heriot-Watt University, Edinburgh, EH14 4AS, UK
[5]Optoelectronics Research Centre, University of Southampton, Highfield, Southampton, Hampshire. SO17 1BJ
[6]Institute for Micro and Nano Systems, University of Edinburgh, Alexander Crum Brown Road, Edinburgh EH9 3FF, UK
[*]R.E.Warburton@hw.ac.uk

ABSTRACT

Recording processes and events that occur on sub-nanosecond timescales poses a difficult challenge. Conventional ultrafast imaging techniques often rely on long data collection times, which can be due to limited device sensitivity and/or the requirement of scanning the detection system to form an image. In this work, we use a single-photon avalanche detector array camera with pico-second timing accuracy to detect photons scattered by the cladding in optical fibers. We use this method to film supercontinuum generation and track a GHz pulse train in optical fibers. We also show how the limited spatial resolution of the array can be improved with computational imaging. The single-photon sensitivity of the camera and the absence of scanning the detection system results in short total acquisition times, as low as a few seconds depending on light levels. Our results allow us to calculate the group index of different wavelength bands within the supercontinuum generation process. This technology can be applied to a range of applications, e.g., the characterization of ultrafast processes, time-resolved fluorescence imaging, three-dimensional depth imaging, and tracking hidden objects around a corner.

Data Processing - Algorithm B

The SPAD camera records three dimensional (space -2D and time 1D) data of an observed event. Each SPAD element of the array indeed operates in time-correlated single-photon counting (TCSPC) mode and produces a histogram with the photon arrival times. At a first instance, the data can simply be visualized as movie, where each frame shows the spatially resolved photon flux density for a given time bin, defined as the temporal resolution of the TCSPC electronics. However, the quality of the data produced this way is negatively affected by the limited spatial resolution of the available arrays (32x32 pixels in the reported experiments) and by the low signal to noise ratio resulting from short acquisitions (the signal to noise ratio increases with the acquisition time). Depending on the specific imaging problem considered, it is however possible to devise strategies to enhance the quality of the data. Here we report on a data processing routine we have implemented to enhance the acquire data and that can be applied when the spatially resolved dynamics can be mapped from its 3D space to a 2D one.
We consider that for pulses propagating into a fiber, the pulse dimension in the direction orthogonal to the pulse propagation can be ignored. This is a direct consequence of the finite spatial resolution of the device, that is not able to sample the spatial scale of the fiber length (in the tens of centimetres range) and of the fiber width (in the micrometer range). Furthermore, for the single mode fiber case, we can safely assume that no interesting dynamics is occurring along the fiber transverse direction. We also assume the pulse path in the three dimension is a simple curve, i.e. no cross points occur (in space and time). In this case, it is possible to construct a two dimensional array that describes the photon flux density in the distance-time space (2D). The routine starts by digitalizing the pulse path into a curve $s(t_i) = \{x(t_i), y(t_i)\}$, e.g. sampling the x-y coordinates of the local maximum in the photon flux density for each step of the evolution. Note that, this procedure still works when the projection of the pulse curve in the spatial 2D space (i.e. after summing of the photon flux densities in the time coordinate) is not a simple curve. After this first step,it is possible to introduce a new coordinate, the distance d, as $d(t_1) = 0$ and:

$$d_i = \sum_{i=2:N} \sqrt{[(x(t_i) - x(t_{i-1}))^2 + (y(t_i) - y(t_{i-1}))^2]}, \tag{1}$$

where N is the number of time bins in the acquisition. Once this d is properly manipulated so that is a normal curve as a function of the discretization, a new data matrix can be constructed starting from the original data cube, where the photon flux density is mapped to a 2D array, function of the time and the distance. The relevant information contained in the original data cube can therefore be represented by a 2D image, as shown in Fig. xx A. Although not clearly evident, Fig. xx A shows a large number of skewed separate traces, that are a consequence of the presence of multiple laser pulses within the time windows of the TCSPC electronics. We consider now a set of transformations Σ_j that shift each pulse p_j into the reference frame of its own group velocity:

$$t'_i = t_i - \frac{d_i}{v_g^{(i,j)}} + c_j \tag{2}$$

$$d'_i = d_i, \tag{3}$$

where $v_g^{(i,j)}$ is the $j-th$ pulse group velocity, at the coordinate d_i, and c_j is a parameter such that $c_1 = 0$ and identifies the temporal distance between the $j-th$ and the $j-th+1$ pulses. Assuming that the group velocity is constant upon the propagation, it can be evaluated by considering that in the reference frame of the pulse group velocity all the pulses maxima should be aligned in time, i.e.:

$$t'_i = t_i - \bar{t}_{i,j} + c_j \tag{4}$$

$$d'_i = d_i, \tag{5}$$

where $\bar{t}_{i,j}$ is the position of the $j-th$ pulse photon flux density maximum at distance d_i. With this map, the 2D array takes the shape shown in Fig. xx B and the group velocity can be evaluated as:

$$v_g^{(i,j)} = \frac{d_i}{\bar{t}_{i,j}}. \tag{6}$$

Finally, since each pulse is supposed to have the same group velocity, constant along the propagation, this can be estimated for the specific fiber, and at the specific wavelength, considering the average $v_g = \langle v_g^{(i,j)} \rangle = \langle d_i / \bar{t}_{i,j} \rangle$.
Once the data are in the form shown in Fig. xx B, further assumptions can be made to enhance the final output. We can consider for instance that each pulse has a Gaussian temporal profile, with a duration that does not vary along the path. This assumption is consistent with the experimental setting since the pulses employed in the experiment are much shorter compared to the detector impulse response function, even after few meters of propagation in the fiber. We can therefore replace the data at each distance step with an arbitrary function (e.g. Gaussian) with a width and an amplitude evaluated from a fit of the data.

Furthermore, we can assume that changes on the pulse amplitude along the propagation are, on a short range (pixel to pixel), artifacts due to the limited resolution and by the path digitalization, while on a long range they are a consequence of losses (not visible in the reported experiment). Eliminating the former with a with an appropriate interpolation method, and back transforming into the laboratory frame results in an enhanced 2D output. As a last step of the routine we can map the data back on the 3D space.

Video legends

Video 1: Supercontinuum generation captured by SPAD camera with 450 nm bandpass filter
Video 2: Supercontinuum generation captured by SPAD camera with 500 nm bandpass filter
Video 3: Supercontinuum generation captured by SPAD camera with 550 nm bandpass filter
Video 4: Supercontinuum generation captured by SPAD camera with 600 nm bandpass filter
Video 5: Supercontinuum generation captured by SPAD camera with 650 nm bandpass filter
Video 6: Supercontinuum generation captured by SPAD camera
Video 7: GHz pulse train in fiber captured with SPAD camera
